# Exploring the potential cost-effectiveness of a vocational rehabilitation program for individuals with schizophrenia in a high-income welfare society

**DOI:** 10.1186/s12888-019-2130-7

**Published:** 2019-05-07

**Authors:** Stig Evensen, Torbjørn Wisløff, June Ullevoldsæter Lystad, Helen Bull, Egil W. Martinsen, Torill Ueland, Erik Falkum

**Affiliations:** 1 Department of Research, Division of Mental Health and Addiction, Oslo University Hospital, Oslo, Norway; 2 Department of Infectious Disease Epidemiology and Modelling, Norwegian Institute of Public Health, Oslo, Norway; 3 Department of Clinical Medicine, University of Oslo, Oslo, Norway; 4 Department of Psychology, University of Oslo, Oslo, Norway; 5 Department of Health Management and Health Economics, University of Oslo, Oslo, Norway

**Keywords:** Schizophrenia, Vocational rehabilitation, Quality adjusted life years (QALY), Cognitive behaviour therapy, Cognitive remediation, Incremental cost-effectiveness ratio

## Abstract

**Background:**

Over the past decades research has shown that employment has a positive impact on quality of life, global functioning and recovery in individuals with schizophrenia. However, access to vocational rehabilitation services for this group is limited and unemployment rates remain high. In this study we explore the potential cost-effectiveness of a novel vocational rehabilitation program (The Job Management Program – JUMP) earmarked for individuals with schizophrenia in Norway.

**Methods:**

The JUMP study was a vocational rehabilitation program augmented with either cognitive behaviour therapy or cognitive remediation. In addition to the JUMP protocol, we extracted treatment cost data from comprehensive and mandatory health and welfare registers. The costs over a two-year follow-up period were compared with the costs over the two-year period prior to inclusion in the study. We also compared the cost-effectiveness of JUMP with a treatment as usual group (TAU).

**Results:**

We identified significant reductions in inpatient services in the JUMP group, both for those who obtained employment and those who did not. Significant reductions were also found in the TAU group, but adjusted for baseline differences the total cost for JUMP participants were € 10,621 lower than in the TAU group during the follow-up period.

**Conclusion:**

In addition to supporting individuals with schizophrenia obtain employment, JUMP appears to have reduced the reliance on mental health services, which should be of interest to stakeholders.

**Trial registration:**

ClinicalTrials.gov Identifier: NCT01139502. Retrospectively registered on 6 February 2010.

## Background

Employment is associated with improved quality of life and global functioning, and is an important part of recovery for individuals with schizophrenia [1–4]. Nevertheless, unemployment rates remain high [5, 6], and in Norway only 10% of the population with schizophrenia is employed [7]. This rate is stable across age groups, and the transition rate from disability benefits into employment is close to zero [8].

Supported employment programs have proven superior compared to sheltered workshops or day service programs in supporting individuals with severe mental illnesses attain competitive employment [9–13]. Benefits of employment include social integration, increased quality of life, higher self-esteem, and improved global functioning [4, 14–16]. A few cost-effectiveness analyses have also been undertaken showing a favourable effect of supported employment in terms of increased employment rates and reduced health service costs [10, 17–19]. However, due to large variations in costs of health and welfare services and income levels between countries it is difficult to generalise these results [20]. Thus, before implementation can be recommended, the cost-effectiveness of an intervention needs to be evaluated in its intended setting.

Vocational rehabilitation (VR) services in Norway are primarily provided by enterprises that offer both sheltered work and supported employment. These services are funded through the Norwegian Labour and Welfare Administration (NAV) and are typically combined with welfare schemes such as NAV paying the individuals’ salary for extended periods, or work placement with no salary beyond disability benefits [16, 21]. This practice has been described as the “benefit trap” [8], and is likely contributing to the high unemployment among individuals with schizophrenia in Norway [7, 8, 21]. Due to the ineffectiveness and limited access to these services for individuals with schizophrenia spectrum disorders, the Job Management Program (JUMP) was established as a VR program for this group with the primary aim of supporting participants obtain competitive employment. A secondary aim was to explore the effect of augmenting VR with cognitive behaviour therapy (CBT) or cognitive remediation (CR). At two-year follow-up 21.2% of the participants in the JUMP study had obtained competitive employment. A further 25.3% had work placements in competitive workplaces, and an additional 13.7% had sheltered work. Both intervention groups (CR and CBT) improved on global functioning, self-esteem, neurocognitive functioning and depression during the follow-up period [16, 22, 23].

### Aims

The objective of the current study was to explore the potential cost-effectiveness of the JUMP intervention compared to a treatment as usual (TAU) group in terms of mental health service costs and effectiveness.

## Methods

### The JUMP study

The JUMP study was a multi-site VR program for adults with schizophrenia spectrum disorders conducted in six Norwegian counties. The program provided 10 months of standard VR services in competitive or sheltered workplaces, which involved assessments, writing job applications, and preparing for interviews. When required, participants also practiced skills, and had job tasks adapted to accommodate for difficulties at the workplace. In addition to the standard VR services the JUMP protocol included three add-ons: (1) there was a formalised collaboration between VR enterprises, NAV and the mental health services to ensure coordinated and ongoing support; (2) participants, collaborators, and in some cases employers received psychoeducation on common elements associated with schizophrenia spectrum disorders; and (3) either CR or use of CBT techniques provided by trained employment specialists twice a week for a 6 month period. Three counties were randomised to CR and three to CBT. Participants were given the intervention provided in their catchment area [16, 22, 24].

### Participants

Participants were recruited from within the mental health services, NAV, and through self-referral in the six counties that were involved in the JUMP study. All participants provided written informed consent. Inclusion criteria were: age between 18 and 65; a diagnosis within the broad schizophrenia spectrum disorders (schizophrenia, schizoaffective disorder, psychotic disorder not otherwise specified and delusional disorder) [25]; sufficient understanding of the Norwegian language; and an IQ above 70. Individuals with neurological disorders, head trauma with more than ten minutes of unconsciousness, and medical conditions that interfered with cognitive function were excluded from the study. Also, individuals who displayed high risk of violent behaviour or severe suicidal ideation, and individuals with ongoing alcohol or substance abuse were not permitted to participate [16, 22, 26–29].

**Fig. 1:**
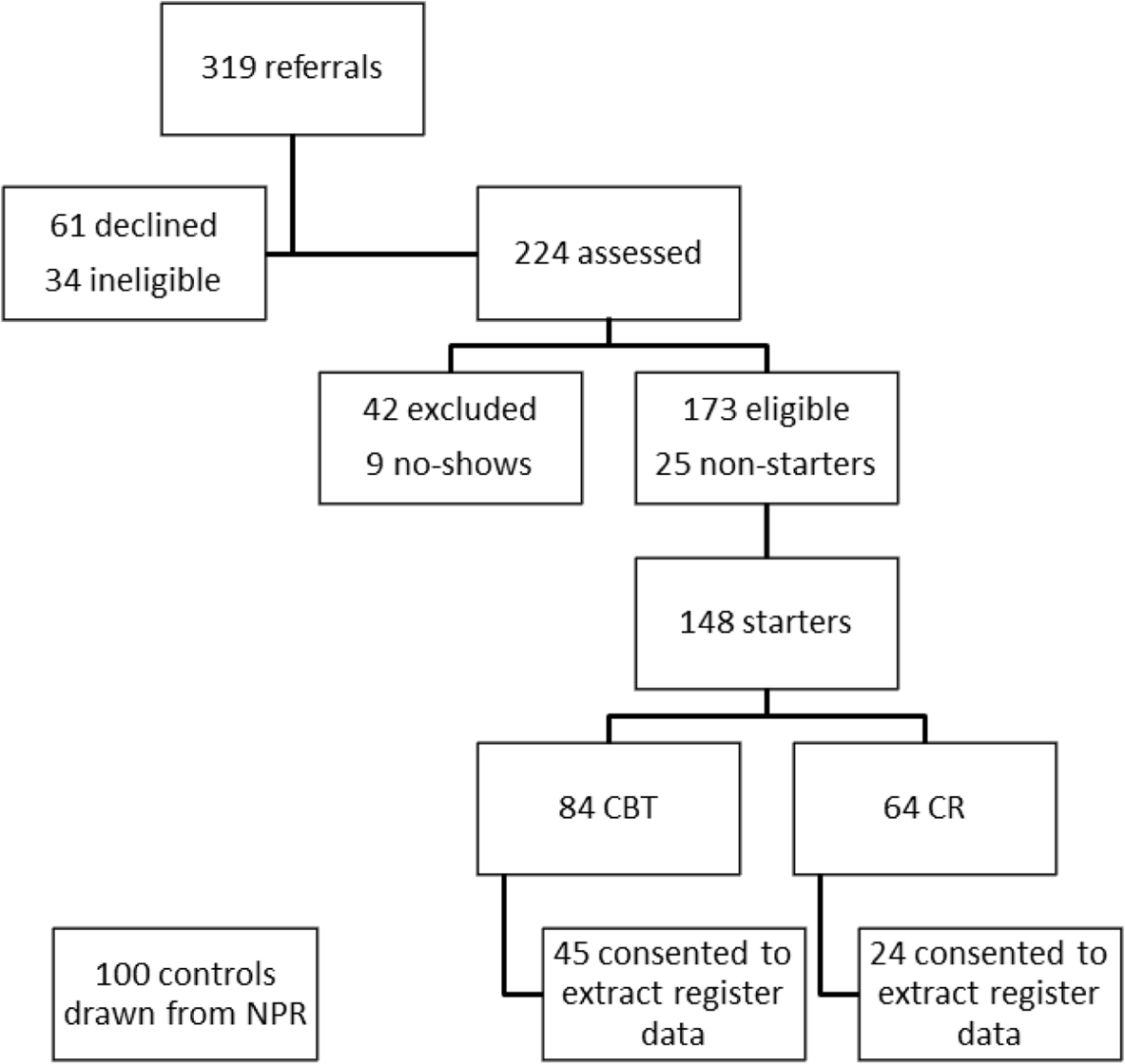
Subject flow in the JUMP study; referrals, starters and participants consenting to cost-effectiveness analysis

A total of 148 participants were included in the JUMP study between August 2009 and March 2012, 84 and 64 respectively allocated to the CBT and CR interventions [16, 28] (Fig. 1).

### Economic evaluation

The economic evaluation was carried out from the perspective of the health and social care system. This included calculating the cost of mental health services, community care services, social security services, medications and primary health care services, in addition to the costs of the CR and CBT interventions. In accordance with the Norwegian Directorate of Health’s guidelines for economic evaluations in the health sector [30], the number of months employed were converted into quality adjusted life years (QALYs) and served as the measure of effectiveness for the cost-effectiveness analysis. Costs were summarised for two 24-month periods: T0 = the last 2 years prior to inclusion in the study and T1 = from inclusion in the study to two-year follow-up. Intervention costs were included in T1.

We examined the intervention costs and health- and welfare costs for T0 and T1. The mental health service costs in the JUMP group were compared to a control group that received treatment as usual (TAU). Effectiveness was measured in terms of QALYs gained during T1 as compared to T0.

### Data sources

Data on services and costs were obtained from comprehensive health and welfare registers: The Norwegian Patient Register (NPR); The Norwegian Prescription Database (NorPD); The Norwegian Health Economics Administration (HELFO); The Individual-based Register of Care Services (IPLOS); and The Norwegian Labour and Welfare Administration’s (NAV) registers on social security benefits, sick leave payments, disability benefits, and competitive employment. The registers are described in detail elsewhere [7]. All data were collected based on the participants’ unique personal identification number. The cost-effectiveness analyses were not part of the original design of the JUMP study, thus a separate consent for extraction of register data after completion of the program was required. Participants were contacted during July – September 2014.

### Intervention costs

The intervention costs included costs of employment specialists, a coordinator from the mental health services at each of the six sites, and overhead costs. Each participant was allocated 6 h of the employment specialists’ time per week, including 2 h of CR or CBT in addition to VR services and collaboration with other involved parties.

### Treatment as usual

The TAU group was drawn from the NPR. Upon approval of extracting data from the NPR, all JUMP participants (*n* = 148) were excluded from the draw based on personal identification numbers. Statisticians at the NPR drew a random sample of 100 individuals with a primary diagnosis of schizophrenia that was matched with the JUMP group on age and sex categories (Table 2). The data were annualised for the period 2010–2012. Only individuals in active treatment in the specialised mental health services (Hospitals, Community Mental Health Centres, and private Psychiatrists and Psychologists with a provider licence) are included in the NPR.

### Currency conversions

All costs were converted to 2015 Norwegian Kroner (NOK) based on the average consumer price index [31]. Costs are reported in €. For currency conversions we used The Central Bank of Norway’s average annual exchange rate for 2015 (8.95 NOK = 1 €) [32].

### Employment data

Employment status for JUMP participants was recorded weekly by employment specialists during the intervention period. Employment status between the end of the intervention period and two-year follow-up were obtained through interviews with the participants and confirmed against NAV’s employee register. Baseline (T0) employment data were obtained through NAV’s employee register. No employment data was available for the TAU group as we did not have approval to merge the NPR with the employment register for this sample. Thus we assumed a stable employment rate of 10.2% for this group based on the national employment rate identified for individuals with schizophrenia in past studies using data from 2012 [7, 8, 33–35].

### Quality adjusted life years

Cost-effectiveness studies commonly use quality-adjusted life-years (QALYs) as a generic outcome measure [36]. QALYs are estimated on the bases of health related quality of life measures. Quality of life measures were not part of the JUMP protocol, thus QALYs were calculated by multiplying months of employment and unemployment with quality of life tariff scores associated with paid employment (0.87) and unemployment (0.79) from the Schizophrenia Health Outcomes (SOHO) study [2]. These scores were based on EuroQol-5D (EQ-5D) scores in individuals who had received 3 years of continuous treatment for schizophrenia. Paid employment was defined as part-time or full-time employment in competitive workplaces. Twenty-one (30.4%) of the JUMP participants (*n* = 69) had paid employment during the two-year follow-up period, and sixteen (23.2%) were employed at the time of the two-year follow-up.

### Analyses

IBM SPSS Statistics version 21.0 [37] and Stata version 13.1 [38] were used for statistical analyses. All tests were two-tailed with a significance level of 5%. In order to examine group differences, baseline comparisons were conducted with Bootstrap Students t-tests or Chi-square tests. Generalized Linear models (GLM) with gamma family and identity link with baseline costs as a covariate were used to compare costs between groups during the two-year follow-up period. Cost data are often skewed as they are always > 0 and the variance is likely to increase with higher expected costs, and thus violate normality assumptions. The GLM approach provides robust estimates of the mean by accommodating for skewness in the data via 3 components: the random component (cost); the systematic component (treatment group); and the link function (relationship between the random and systematic components). In the gamma family the variance of cost is assumed to be proportional to the square of the mean, which gives a better fit than the normal distribution [39, 40]. To adjust for potential bias due to the lack of randomisation, we performed propensity score adjustments with age, sex, and baseline day treatment, inpatient and outpatient care as potential bias variables. The propensity score was included as a covariate in the GLM. In health economic evaluations, it is recommended to perform probabilistic sensitivity analysis to assess the uncertainty of the resulting cost-effectiveness. We performed probabilistic sensitivity analysis on resulting costs and effects and combined these to provide an estimate of the probability of JUMP being cost-effective compared to TAU.

## Results

### Baseline analysis

Sixty-nine (46.6%) participants from the JUMP study consented to extraction of register data for this study. There were no significant differences between those who consented and those who declined on key variables (diagnosis, age, sex, units of DDD of antipsychotic medication, psychotic symptoms, education, previous work experience, employment outcome (Table 1) at two-year follow-up, thus we assume the results are fairly representative for the participants in the JUMP study.

**Table 1:** Comparison on key variables between participants who consented to obtaining register data and participants who declined at two-year follow-up

	Consent (*N* = 69)	Declined (*N* = 79)	Test Statistics	Group comparison (*p*)
Diagnosis				
Schizophrenia	87.0%	89.9%		
Schizoaffective disorder	8.7%	6.3%	*Χ*^*2*^ (4, n = 148) = 1.68	Ns
Psychosis NOS	1.4%	2.5%		
Delusional disorder	2.9%	1.3%		
Age, mean (SD)	33.2 (7.7)	32.6 (8.2)	*t* (n = 148) = 0.40	Ns
Gender, male (%)	45 (65.2%)	58 (73.4%)	*Χ*^*2*^ (1, n = 148) = 1.17	Ns
Education, highest completed		32.9%		
Primary school	30.4%	38.0%		
High school	29.0%	13.9%	*Χ*^*2*^ (5, n = 148) = 8.27	Ns
Trade school	8.7%	8.9%		
College	21.7%	6.3%		
University	7.2%			
Not completed primary school	2.9%			
Units of DDD^b^ main anti-psychotic, mean (SD)	1.1 (0.9)	1.1 (0.7)	*t* (140) = 0.83	Ns
Duration of illness, mean years (DOI) (SD)	8.0 (6.7)	6.5 (6.1)	*t* (143) = 1.42	Ns
Previous work experience, mean months (SD)	64.15 (65.32)	66.69 (73.82)	*t (n = 146) = − 0,22*	Ns
Psychotic Symptoms (PANSS total) (SD)	56.23 (15.35)	60.19 (15.34)	*t (n = 141) = − 1.52*	Ns
Employment outcome				
Competitive employment	23.2%	21.4%		
Work placement	34.8%	18.6%		
Sheltered work	13.0%	15.7%	*Χ*^*2*^ (3, *n* = 139) = 5.87	Ns
Unemployed	29.0%	44.3%		

^b^ Defined daily Dose (DDD)

All JUMP participants had primary diagnoses in the schizophrenia spectrum (87% schizophrenia) while subjects in the TAU group had schizophrenia as their primary diagnosis. There were no significant differences in sex or age (Table 2).

**Table 2:** Comparison on key baseline characteristics between JUMP and TAU

	JUMP (*N* = 69)	TAU (*N* = 100)	Test Statistics	Group comparison (*p*)
Age, mean (SD)	33.2 (7.7)	34.9 (9.1)	*t* (*n* = 169) = − 1.32	Ns
Gender, male (%)	45 (65.2%)	65 (65.0%)	*Χ*^*2*^ (1, n = 169) = 0.001	Ns
Diagnosis				
Schizophrenia	87.0%	100.0%		.
Schizoaffective disorder	8.7%		*Χ*^*2*^ (3, n = 169) = 13.78	003
Psychosis NOS	1.4%			
Delusional disorder	2.9%			

### Costs and service utilization

The mean cost of the JUMP intervention was € 9131 (SD 2123) per participant. The mean duration of the intervention was 26.52 weeks (SD 5.89).

There were significant reductions in inpatient services from T0 to T1 for both the JUMP participants (mean = € -90,944; 95CI -137,781, − 46,165; *p* < .012) and the TAU group (mean = € -78,116; 95% CI -126,941, − 33,275; *p* = .010). Combining inpatient and outpatient costs gives a mean reduction of € 87,809 in the JUMP group and € 76,386 in the TAU group (median: € -1335 and € 13,263 respectively). The reduction in the JUMP group was significant both for those who gained paid employment (*n* = 21; mean = € -80,776; 95% CI -140,112, − 21,467; p = .010) and for those who had work placement or sheltered work (*n* = 42; mean = € -90,885; 95% CI -153,873, − 27,897; *p* = .006). No significant changes were found for other health or welfare costs. Mean and median costs for each 24 - month period; T0 and T1 (inclusive of intervention costs) are detailed in Table 3.

**Table 3:** Mean and median specialised mental health costs (€ 2015) for 24-month period at T0 and T1 (n JUMP = 69, n TAU = 100), and mean primary health and social care cost at T0 and T1 (JUMP)

	JUMP T0		JUMP T1		TAU T0		TAU T1	
	€	SD	€	SD	€	SD	€	SD
Inpatient services	126,493	235,513	35,549	96,502	168,915	330,530	90,798	177,827
Outpatient services	13,853	15,128	16,988	24,656	6251	10,917	7982	12,851
Total specialised mental health (mean)	140,345	236,516	66,519	102,942	175,165	331,739	98,779	179,602
Total specialised mental health (median + range)	30,590	1,067,387	29,255	642,030	7658	1,082,098	20,920	1,025,296
Primary health care	515	746	426	552				
Community care incl. Accommodation	56,387	110,280	60,690	108,645				
Medications	2999	3443	2958	3329				
Social security	32,172	16,795	41,934	11,154				
Intervention cost			13,982	3154				
Total	232,419	263,821	172,527	148,587				

In terms of utilisation of mental health services, the JUMP group had significantly more outpatient visits during both T0 and T1 compared to TAU. At T1 the JUMP group had significantly fewer days of hospitalisation than the TAU group (Table 4). Bootstrap paired samples t-tests revealed significant reductions in inpatient days for both JUMP (mean − 63.0; 95% CI -96.2, − 33.3; p = .006) and for TAU (mean − 53.8; 95% CI -87.5, − 23.1; *p* = .004). We found no significant differences in change scores between JUMP and TAU through bootstrap independent samples t-test (mean 9.2; 95% CI -39.9, 58.5; *p* = .71).

**Table 4:** Comparison of mental health service utilization between JUMP and TAU at T0 and T1

	JUMP (*N* = 69)	TAU (*N* = 100)	Test Statistics	Group comparison (*p*)^a^
T0				
Days of hospitalisation T0 (SD)	88.5 (164.8)	118.4 (231.6)	*t* (n = 169) = .922	Ns
Days of day treatment T0 (SD)	.54 (2.5)	.28 (1.7)	*t* (n = 169) = .784	Ns
Outpatient visits T0 (SD)	48.5 (53.4)	22.0 (39.7)	*t* (n = 169) = −3.708	<.001
T1				
Days of hospitalisation T1 (SD)	25.5 (69.3)	64.6 (126.5)	*t* (n = 169) = 2.336	.021
Days of day treatment T1 (SD)	1.1 (8.0)	.51 (2.9)	*t* (n = 169) = .687	Ns
Outpatient visits T1 (SD)	59.9 (89.6)	28.1 (45.65)	*t* (n = 169) = −3.027	.003

^a^ Based on bootstrap t-tests with 1000 samples

We performed a generalized linear regression with costs at T0, group (JUMP/TAU) and the propensity score as covariates using gamma family and identity link to examine group differences in total costs at T1 (Table 5). We tested the assumptions with a linktest which was non-significant, indicating that the assumptions are reasonable. Total mean costs for the JUMP group (inclusive of intervention costs and adjusted for baseline differences) were € 10,621 lower than for TAU (95% CI: -29,979, 8735; *p* = .282). Costs at T0 was a significant predictor of costs at T1 (Table 5).

**Table 5:** Effect of group (TAU vs JUMP), baseline costs and propensity score (age, sex, and baseline day treatment, inpatient and outpatient care) on treatment costs at T1 (GLM)

	Coeff	95% CI	P
Group – TAU vs JUMP	−10,621.64	−29,979, 8735	0.282
Baseline cost	.26	.18, .35	< 0.001
Propensity score	65,033.19	−24,799, 154,865	0.156

### Cost-effectiveness

**Fig. 2:**
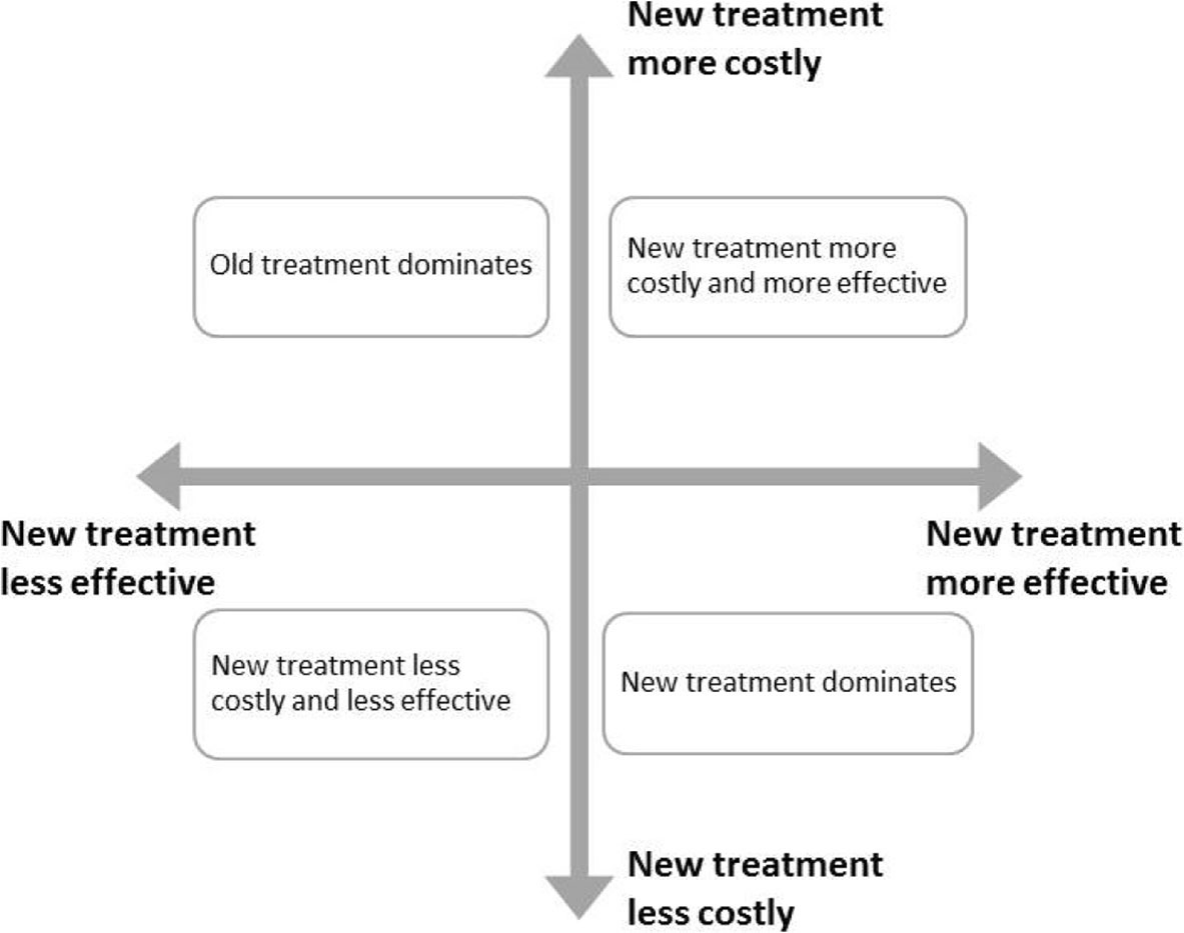
The cost-effectiveness plane [48]

When assessing whether a program is cost-effective it is useful to use the cost-effectiveness plane (Fig. 2). If the mean value of the new program is more effective and less costly than the reference program, it is cost-effective and is typically denoted as a dominant strategy. Likewise, if the new program is less effective and more costly it is dominated [41]. New programs in the two other quadrants of the plane are evaluated against a cost-effectiveness threshold value suggested by the Norwegian Directorate of Health which is € 62,000 per QALY gained [42].

In terms of exploring the cost effectiveness of an intervention the effect is usually estimated as a general measure of gained health related quality of life from the new intervention compared to existing treatment, which is converted into quality adjusted life years (QALY).

The mean months of competitive employment in the JUMP group were 3.10 (*N* = 69, SD 7.27) at T0 and 4.30 (N = 69, SD 7.33) at T1 with corresponding QALYs being .8003 (SD .024) at T0 and .8043 (SD .024) at T1. Thus the incremental QALYs for competitive employment were .004 (SD .026; 95%CI −.002, .010) in the JUMP group, while we assumed a stable 10.2% employment rate in the TAU group.

Dividing the aggregate difference in average specialised mental health service costs (€ 10,621) by the aggregated improvement in QALYs yields a negative incremental cost-effectiveness ratio, which places the JUMP group in the bottom right quadrant of the cost-effectiveness plane (more effective and less costly) (Fig. 2). Although the difference is not significant there is a trend towards the JUMP program being more cost-effective. This is also the case if using incremental months of work as the effect measure. JUMP generated more months of employment than TAU at a lower cost. Probabilistic analyses indicate an 85% probability that JUMP is cost-effective compared to TAU.

## Discussion

This study found reduced mental health service costs during a novel vocational rehabilitation program (JUMP) for individuals with schizophrenia spectrum disorders in Norway. Compared to a TAU group the mean mental health costs, adjusted for baseline differences (T0), were € 10,621 lower in the JUMP group (inclusive of intervention costs) during the two-year follow-up period (T1). Due to skewed data we also provided median costs for specialised mental health services. The median costs for the JUMP group reduced slightly from T0 to T1, while the median cost for the TAU group increased by € 13,261. This is further evidence in favour of the JUMP intervention [43]. A large mean reduction in mental health service use was also identified in the TAU group. One potential explanation for this reduction may be that only patients in active treatment within the specialised mental health services are registered in the NPR. Hence, the reduced reliance on mental health services in the TAU group is likely an effect of therapeutic interventions beyond our control. The service use variation was also greater in the TAU group with the median group difference being greater than the mean.

We also found that competitive employment during 24 months in the JUMP group increased from a mean 3.1 months at T0 to 4.3 months at T1. When including all types of employment the mean months of employment at T1 was 15.14 months. The assumed duration of competitive employment in the TAU group (based on previous studies [7, 8, 33–35]) was 2.35 months. The incremental QALYs related to competitive employment were .006 higher in the JUMP group than the TAU group at T1. Despite the costly intervention, mental health service costs were reduced in the JUMP group and there was an increased effect measured in both QALYs and months worked.

The cost reductions in the current study were driven by a large reduction in inpatient care at T1. Similar reduced reliance on inpatient care for individuals who gain employment have also been documented in other studies [44–46]. A commonly used argument for such results is that work in itself facilitates symptom improvement and enhanced self-esteem [4, 14, 15, 46], which in turn is likely to reduce hospitalisations. This is probably one of several factors in play, and some studies have displayed somewhat different results. In a study comparing supported employment with traditional VR across six European cities, Knapp and colleagues found that participants in the supported employment group utilised significantly less inpatient services than participants in the traditional VR group during the first 12 months of the study, while there was no difference during the six months thereafter [19]. In contrast, the current study found the reliance of inpatient care to remain significantly lower than in the TAU group throughout the 24 - month follow-up period. In a recent study comparing supported employment to traditional VR in Norway, Reme and colleagues found that supported employment did not have a significant effect on inpatient care [47]. Notably this study had broader inclusion criteria, thus the results are not directly comparable.

An interesting point when comparing the current study with supported employment studies is that we found no significant differences between those who gained competitive employment and those who had work placements or sheltered work in terms of reliance on mental health services. This is important as it indicates that vocational rehabilitation programs for individuals with schizophrenia can have positive health effects even when employing a broader definition of work than is normally the case in supported employment studies.

The reasons for the reduced inpatient care in the current study are likely multifaceted. One explanation may be that JUMP participants increased the use of outpatient mental health services, which probably contributed to fewer hospitalisations. Another important factor could be the close follow-up and two weekly CBT or CR sessions the participants received from the trained employment specialists. In addition to perhaps being therapeutic in itself, the close follow-up enabled the employment specialists to intervene, either directly or by alerting clinicians, if they observed signs of symptom increase.

Past cost-effectiveness studies have primarily examined programs where competitive employment has been the only employment outcome measured. Although competitive employment was the ultimate aim of the JUMP study, work placements and sheltered work were also considered a success in accordance with the Scandinavian model of vocational rehabilitation. It should be noted that the use of work placements and sheltered work was an important factor that enabled 77% of the participants in the JUMP study to maintain their job during the ten-month intervention period [22].

Apart from the reduction in mental health service costs all costs except social security remained stable between T0 and T1. We believe the change was primarily due to participants having their entitlements revised upon inclusion in the project. Consequently, many were shifted from a disability pension to a work assessment allowance, which provided them with a higher benefit. At two-year follow-up 16.4% of all JUMP participants had transitioned from disability benefits to paid employment as their primary source of income. Any long-term effect on social security costs will be explored in the forthcoming five-year follow-up of the JUMP study.

### Strengths and weaknesses

A major strength of this study was the use of comprehensive and compulsory health- and welfare registers, which provided detailed records of participants’ societal costs over a period of four years. The main limitation of the study is the lack of a randomised control group. Due to recruitment problems we were forced to abandon the original design of including a control group and thus drew a TAU group from the NPR for this study. Although the TAU group consisted of a random selection, there were large mean differences in resource utilisation between the TAU and JUMP groups at baseline. This may indicate that there were clinical differences between the two groups but as we do not have any clinical information about the TAU group, we are unable to determine if such differences were, in fact, present. By controlling for baseline differences, the effect of this difference is likely to have been reduced. Due to the lack of a control group receiving VR only, we were unable to disentangle the effects of the CR and CBT interventions. Also, no direct measure of health related quality of life was included in the design of the study; hence tariff scores from the literature were used. Although this method is commonly used in health economic evaluations where measures of health-related quality of life are not available, the validity of this estimation should be interpreted with caution as health related quality of life can be influenced by a number of factors [20]. The lack of employment and quality of life data for the control group is also a limitation that affects the validity of the results. Another limitation is that less than half of the participants in the JUMP study consented to extraction of register data. There were, however no significant differences on key variables between those who consented and those who did not. Finally, the high intervention costs of the JUMP study as well as Norway’s high treatment costs and strong welfare system affect the generalisability of our results.

## Conclusion

The current study identified non-significant cost reductions and improvements in QALYs among JUMP participants compared to TAU. The main cost-effect was driven by reduced inpatient services. The reductions in mental health costs were similar both for those who gained competitive employment and those who had work placements or sheltered work. This indicates that JUMP was a beneficial mental health treatment approach, which should be of great interest to service providers. This is particularly relevant given the current need in Norway to reduce the substantial economic burden of unemployment/risk of unemployment due to severe mental illness [8]. Although the point estimate indicates that JUMP both increased quality adjusted life years (QALY) and reduced costs, there is uncertainty concerning both variables.

## Abbreviations

CBT: Cognitive behaviour therapy
CR: Cognitive remediation
DDD: Defined daily dose
DSM-IV: Diagnostic and statistical manual - iv
EQ-5D: EuroQol – 5 dimensions
GLM: Generalized linear model
HELFO: The Norwegian Health Economics Administration
HoNOS: Health of the Nation Outcome Scales
IBM SPSS: IBM (International Business Machines) Statistical analysis software package
IPLOS: The Individual-based Register of Care Services
IQ: Intelligence quotient
JUMP: Job management program
NAV: The Norwegian Labour and Welfare Administration
NOK: Norwegian kroner
NorPD: The Norwegian Prescription Database
NPR: The Norwegian Patient Register
QALY: Quality adjusted life years
TAU: Treatment as usual
VR: Vocational Rehabilitation
